# New Modes of Thought Based upon the New Materialism and the New Pantheism: Including a Tribute to Edward Drinker Cope

**Published:** 1901-12

**Authors:** 


### Bibliography.

New Modes of Thought based upon the New Materialism and the New Pantheism: Including a Tribute to Edward Drinker Cope. By C. T. Stockwell, Author of "The Evolution of Immortality." James H. West Company, Boston.

Those who had the pleasure of reading Dr. Stockwell's "Evolution of Immortality" will not need to be urged to procure this more recent publication. It is impossible to notice a work of this character in a profesional journal as it should be reviewed. It belongs to a range of vision not adapted to the ordinary practical mental activities; but yet even here, to the man or woman who can rise above this dead level and allow the mind to delve into the deeper philosophical thought, this work of Dr. Stockwell will be most welcome.

The reason for this publication is outlined in the following paragraph: "These papers were written with no thought or purpose other than to suggest discussion by the literary club for which they were prepared. Shortly after their first reading, however, they found a larger audience through the columns of the *Springfield Republican.* Almost immediately there sprang up a demand for their publication."

That such a demand was made is not surprising, for it is rare to observe so much valuable thought condensed in an equal compass. So much is this the case, that the reader will find it difficult to select any special chapter or paragraph more worthy of notice than another.

Perhaps what the author denominates as "A Thought Summit" very nearly expresses his dominant thought. "The conclusion seems to be growing that, in the evolutionary forces of all nature, mind, or the mental principle, is the sovereign, the dominant force, rather than the structural, the mechanical, the physical, or even that of inheritance and environment;" and further he writes, "That an organism can in time free itself from its inheritance by choosing or creating or reacting upon its environment, and thus climb in the scale of being by virtue of its own intrinsic and inherent forces, is now claiming a larger place in the thought of the world than formerly."

The amplification of this view naturally leads up to the consideration of the governing force of the universe. The author discusses the ether problem, and brings to the support of his theories some of the ablest scientists and thinkers that the nineteenth century has produced. He says of this theory, "In the ether, and in the wave-theory of the structure of the atoms, man is beginning to think he can see, with the eye of science, real substance in, at least, its mechanical or physical and mathematical aspect; and in using the word 'substance' nothing else is implied than that which is commonly understood by the terms 'God,' 'Reality,' 'Spirit,' and so forth."

The author, starting with these premises, naturally and inevitably drifts to the consideration of the "over soul" or God; and we therefore find him logically affirming "we must start with God, at the very outset, as the first principle in even the physical and mechanical world. In the physical, in the mechanical, in the 'grossest' material world of phenomena, as well as in the psychical world, we see but different aspects, equally divine, of one and the same thing; and this One is nothing else than God,—God embodied in the ether."

He further says, "Everything is dynamic, animated, 'quick with living powers, burning with intelligence, glowing with passion, throbbing with emotion, crowded with intentions.' This view leaves no room whatever in the whole universe for death or dead matter. Death is dead. It is illusion."

The second part is devoted to "the New Pantheism," but space will not permit following the author through his interesting argument. Whether it will be accepted by all is very doubtful, and to the reviewer it seems questionable whether the human mind is capable, with our present knowledge, of sounding the great depths hidden away in the immensity of the physical universe. Science has only skimmed the surface of things, and, while it is a universal duty to endeavor to solve the mystery of creation, it is a question whether the solution ever will be given to finite intelligence.

The author closes with an appropriate tribute to one of the greatest scientists the nineteenth century produced,—Edward Drinker Cope.

Whatever criticism may be made of this book of Dr. Stockwell, no one can rise from its reading without feeling his mentality invigorated and his spiritual nature strengthened.

